# Factors Associated with Mental Health Results among Workers with Income Losses Exposed to COVID-19 in China

**DOI:** 10.3390/ijerph17155627

**Published:** 2020-08-04

**Authors:** Xin Li, Peixin Lu, Lianting Hu, Tianhui Huang, Long Lu

**Affiliations:** School of Information Management, Wuhan University, Wuhan 430072, China; XinLi2020@whu.edu.cn (X.L.); Lupx@whu.edu.cn (P.L.); LiantingHu@whu.edu.cn (L.H.); TianhuiHuang@whu.edu.cn (T.H.)

**Keywords:** COVID-19, depression, anxiety, insomnia, distress, income losses

## Abstract

The outbreak and worldwide spread of COVID-19 has resulted in a high prevalence of mental health problems in China and other countries. This was a cross-sectional study conducted using an online survey and face-to-face interviews to assess mental health problems and the associated factors among Chinese citizens with income losses exposed to COVID-19. The degrees of the depression, anxiety, insomnia, and distress symptoms of our participants were assessed using the Chinese versions of the Patient Health Questionnaire-9 (PHQ-9), the Generalized Anxiety Disorder-7 (GAD-7), the Insomnia Severity Index-7 (ISI-7), and the revised 7-item Impact of Event Scale (IES-7) scales, respectively, which found that the prevalence rates of depression, anxiety, insomnia, and distress caused by COVID-19 were 45.5%, 49.5%, 30.9%, and 68.1%, respectively. Multivariable logistic regression analysis was performed to identify factors associated with mental health outcomes among workers with income losses during COVID-19. Participants working in Hubei province with heavy income losses, especially pregnant women, were found to have a high risk of developing unfavorable mental health symptoms and may need psychological support or interventions.

## 1. Introduction

At the end of December 2019, the Chinese city of Wuhan reported a novel pneumonia caused by coronavirus disease 2019 (COVID-19), an infectious disease caused by an acute severe respiratory syndrome coronavirus, which is rapidly spreading both domestically and internationally [1,2]. On 30 January 2020, the World Health Organization (WHO) held an emergency meeting and declared the worldwide COVID-19 outbreak a public health emergency of international concern [3]. The emergence and rapid increase in the number of COVID-19 cases has posed and continues to pose complex challenges for global research, public health, and medical communities [4,5]. As of 1 June 2020, there were more than 6.15 million confirmed cases of COVID-19 across more than 215 countries and regions, including more than 372,130 deaths.

With the rapid spread of COVID-19, the local government in Wuhan immediately adopted a city closure policy, encouraging citizens to work at home and teach online, and shut down non-essential services to mitigate the impact and risks of the disease. Then, the governments of other provinces with low numbers of infected people in China and many other countries around the world entered states of emergency for the health response and issued a series of policies, including ordering citizens (regardless of having symptoms of infection or not) to self-isolate at home, and maintaining social distance from other people. However, concerns have arisen about the potential psychological impact of these measures [6,7,8]. 

Studies proved that COVID-19 has caused a high prevalence of mental health problems in China [8,9,10,11,12] and other countries around the world [13,14,15,16]. Some researchers have attempted to understand the outbreak of this novel coronavirus from a global health perspective [17,18,19]. However, most studies focused on the psychological effects of people who were infected with COVID-19, medical workers, or people in specific regions [10,11,12,13,14,15,20]. Studies showed that the economic impact caused by severe acute respiratory syndrome (SARS) will produce psychological morbidities in individuals who are directly or indirectly exposed to life-threatening situations [21]. The occurrence of such psychological morbidities among workers can impact their daily functions and lead to immediate economic and physiological consequences, such as lost job productivity, depression, and anxiety [22,23]. 

To the best of our knowledge, no previous study focused on mental health problems among people with income losses caused by COVID-19. To address this gap, the aim of our study was to evaluate the mental health of Chinese workers with income losses exposed to COVID-19 by quantifying the degrees of depression, anxiety, insomnia, and distress, and analyze the potential risk factors related to these symptoms. In this study, besides age, sex and other demographic characteristics, participants from Hubei province and outside Hubei province were taken as the research objects for comparison of regional differences. The ultimate goal of this study was to assess the mental health burden of people with income losses during COVID-19 and to provide guidance for the promotion of mental well-being among this population.

## 2. Materials and Methods 

### 2.1. Design and Participants

This was a cross-sectional study conducted using an online survey and face-to-face interviews to assess mental health problems and their associations with income losses among Chinese citizens who were exposed to coronavirus disease 2019 (COVID-19) from 25 April to 9 May 2020. 

Eligibility criteria included (i) currently living in China, (ii) aged 18 years or older, and (iii) with income losses caused by COVID-19. Participants were encouraged to participate in online surveys or complete offline questionnaires. A total of 421 of 600 contacted individuals completed the survey for a participation rate of 70.2%, and 23 people with no loss of income were excluded from the study. The final sample included 398 respondents, with a response rate of 66.3%. This study was approved by the Ethics Committee and Institutional Review Board of Wuhan University, Wuhan, China (Ref: 20200411), and conducted in accordance with the ethical guidelines of the Declaration of Helsinki of the World Medical Association. All data were deidentified before being provided to the investigators. Consent from each participant was obtained at the beginning of the survey.

### 2.2. Questionnaire

The questionnaire consisted of 37 factors to record demographic indicators and symptoms of depression, anxiety, insomnia, and distress caused by COVID-19 of the participants (See Appendix A).

#### 2.2.1. Covariates

The following demographic data were included in this study: sex (male or female), age (18–25, 26–30, 31–40 and >40 years old categories), educational level (<undergraduate, college, masters, and higher), marital status (married and other including unmarried, widowed, and divorced), working location (Hubei province, and outside Hubei province), loss of income caused by COVID-19 (light, middle, or heavy, >0% to 25%, 25–50%, and >50% less than pre-epidemic income, respectively), and place of residence (urban or rural).

#### 2.2.2. Mental Health Problems

Mental disorders, including depression, anxiety, insomnia, and distress, caused by COVID-19 were assessed in our study by Chinese versions of validated measurement tools [24,25,26,27]: the Patient Health Questionnaire-9 (PHQ-9; the total score ranged from 0 to 27) [24], the Generalized Anxiety Disorder-7 (GAD-7; the total score ranged from 0 to 21) [25], the Insomnia Severity Index-7 (ISI-7; the total score ranged from 0 to 28) [26], and the revised 7-item Impact of Event Scale (IES-7; the total score ranged from 0 to 28) [27]. The response options are: 3 = nearly every day, 2 = more than half the days, 1 = several days, and 0 = not at all for PHQ-9 and GAD-7; 4 = always, 3 = often, 2 = sometimes, 1 = rare, and 0 = never for ISI-7 and IES-7. The total scores of these survey scales are interpreted as follows: PHQ-9, extremely severe (22–28), severe (15–21), moderate (10–14), mild (5–9), and normal (0–4) depression; GAD-7, severe (15–21), moderate (10–14), mild (5–9), and normal (0–4) anxiety; ISI-7, severe (22–28), moderate (15–21), subthreshold (8–14), normal (0–7) insomnia; and IES-7 severe (22–28), moderate (15–21), subthreshold (8–14), and normal (0–7) distress. The cutoff score for detecting possible major symptoms of depression, anxiety, insomnia, and distress caused by COVID-19 are 10, 10, 15, and 15, respectively. A higher score indicates participants with greater self-reported severe symptoms [24,25,26,27]. 

The psychometric properties and internal reliabilities of the 4 scales have been previously confirmed in Chinese populations [24,25,26,27]. In [24], statistical tests were performed to determine the reliability and validity of PHQ-9. Results showed that the internal consistency value of PHQ-9 was 0.854 and the test–retest reliability value of PHQ-9 was 0.873, proving the PHQ-9 is a valid and reliable tool to evaluate depression in Chinese people. He [25] tested the reliability and validity of Chinese version of GAD-7. The results show that the Cronbach ‘α coefficient of GAD-7 is 0.898, and the test–retest reliability coefficient is 0.856, proving the Chinese version of GAD-7 has good reliability and validity in the application of evaluating anxiety. Doris S.F. Yu [26] tested the reliability and validity of Chinese version of ISI-7, finding that Cronbach’s alpha of the Chinese version of the ISI-7 was 0.81, with item-to-total correlations in the range of 0.34–0.67. In [27], Chan reported that the Cronbach ‘α coefficient of IES-R is 0.89, which proved the IES-R is a valid and reliable tool to evaluate distress among Chinese people. In our study, the Cronbach’s alpha coefficient of our questionnaire is 0.97. The Cronbach’s alpha coefficients of the Chinese versions of PHQ-9, GAD-7, ISI-7 and IES-7 were 0.920, 0.945, 0.879 and 0.909, respectively.

### 2.3. Statistical Analysis

First, we used descriptive statistics to describe the socio-demographic characteristics of these participants. Second, the prevalence rates of depression (PHQ-9 score ≥ 5), anxiety (GAD-7 score ≥ 5), insomnia (ISI-7 score ≥ 8), and distress (IES-7 score ≥ 8) were estimated. Finally, multivariable logistic regression models were used to explore factors associated with depression, anxiety, insomnia, and distress among workers with income losses exposed to COVID-19 in China, and the associations between risk factors and outcomes are presented as adjusted odds ratios (aORs) with a 95% confidence interval (CI), after adjustment for confounders, including sex, age, marital status, educational level, working position, place of residence, degrees of income losses. Data analysis was performed by SPSS statistical software (version 25.0, IBM Corp., Armonk, NY, USA,), with *p*-values < 0.05 indicating statistical significance. The significance level was set at α = 0.05, and all tests were two-tailed.

## 3. Results

### 3.1. Demographic Characteristics

As shown in Table 1, the proportion of men to women was close, at 50.5% and 49.5%, respectively, and the proportion of marital status (recoded into married and other including unmarried, widowed, and divorced) was similar to that of sex, at 49.5% and 50.5%, respectively. We classified their income losses caused by COVID-19 as one of the demographic variables. Response options were slightly affected (>0% to 25%), moderately affected (25–50%), and heavily affected (>50%).Table 1 shows that the proportions of light, middle, and heavy income loss (>0% to 25%, 25–50%, and >50% lower income than pre-epidemic income, respectively) caused by COVID-19 were 33.9%, 17.6%, and 48.5%, respectively. As Hubei was most severely affected province by COVID-19 in China, all 398 participants were grouped by their geographic location. The proportions in Hubei province, and places outside Hubei province were 44.2%, and 55.8%, respectively. Most of these participants were aged from 26 to 40 years, lived in urban areas, and had a college degree or above.

### 3.2. Prevalence Rates of Mental Symptoms and Associated Factors

Generally consistent with the existing COVID-19 research results [8,9,10], the prevalence rates of our participants who had symptoms of depression, anxiety, insomnia, and distress cause by COVID-19 were 45.5%, 49.5%, 30.9%, and 68.1%, respectively. As shown in Table 2, multivariable logistic regression analyses showed that, after controlling for covariates, the adjusted odds of depression, anxiety, insomnia and distress were lower among participants who under 30 years (e.g., depression among participants aged 26–30 years: OR = 0.228, 95% CI: 0.097–0.535, *p* < 0.001; depression among participants aged 18–25 years: OR = 0.187, 95% CI: 0.072–0.489, *p* < 0.001) compared with who aged over 40 years, and greater among those working in Hubei province (e.g., depression: OR = 2.647, 95% CI: 1.662–4.217, *p* < 0.001) than outside Hubei province. For the population whose income was heavily affected by COVID-19, they were prone to experiencing mental symptoms of depression, anxiety, and insomnia (e.g., depression among participants with light income losses: OR = 0.215, 95% CI: 0.124–0.371, *p* < 0.001). Those from urban area had lower adjusted odds of depression anxiety, insomnia and distress than those from rural area (e.g., depression: OR = 0.391, 95% CI: 0.226–0.675, *p* = 0.001). At the same time, being married (OR, 3.348; 95% CI, 1.896–5.911; *p* < 0.001) was associated with a greater risk of feeling depressed than being unmarried. 

In sex statistics, we set an additional question (If you are a woman, please indicate whether you are pregnant). In this study, as shown in Table 3, multivariable logistic regression analyses showed that, after controlling for covariates, we found that pregnant women with income losses during COVID-19 were associated with a greater risk of feeling depressed and anxiety (depression: OR = 2.956, 95% CI: 1.208–7.229, *p* = 0.018; anxiety: OR = 3.146, 95% CI: 1.217–6.133, *p* = 0.018) than unpregnant women (Table 3).

### 3.3. Prevalence Rates of Severe Mental Symptoms and Associated Factors

According to Lai, J et al. [10], the cutoff scores for detecting possible major symptoms of depression, anxiety, insomnia, and distress caused by COVID-19 are 10, 10, 15, and 15, respectively. Thus, the prevalence rates of our participants who had severe mental symptoms of depression, anxiety, insomnia, and distress were 19.1%, 21.9%, 7.8%, and 25.9%, respectively. Similar to findings regarding prevalence of mental symptoms, as shown in Table 4, multivariable logistic regression analyses showed that, after controlling for covariates, the adjusted odds of severe symptoms of depression, anxiety, and distress were lower among participants who aged 26–30 years (e.g., severe depression: OR = 0.243, 95% CI: 0.091–0.645, *p* = 0.005) compared with who aged over 40 years, greater among those with heavy income losses than light and middle income losses (e.g., severe depression among participants with light income losses: OR = 0.246, 95% CI: 0.121–0.502, *p* < 0.001), and lower among those from urban area than those from rural area (e.g., severe depression: OR = 0.337, 95% CI: 0.185–0.615, *p* < 0.001). For those working in Hubei province, they were more prone to experiencing severe mental symptoms of anxiety and distress than those working outside Hubei province.

## 4. Discussion

We enrolled 398 respondents and found a high prevalence of mental health symptoms among workers with income losses caused by COVID-19 in China. This latest national sample indicated the prevalence rates of any disorder (excluding dementia), anxiety disorders, and depressive disorders were 16.6%, 7.6%, and 6.9% in China, respectively. Compared with national data, we found much higher prevalence rates of participants with symptoms of depression, anxiety, insomnia, and distress caused by COVID-19, at 45.5%, 49.5%, 30.9%, and 68.1%, respectively. Our findings are consistent with those of previous COVID-19 studies, including a study in mainland China that found that the prevalence of depression as measured during the COVID-19 pandemic was 48.3% [8] and a study in Hong Kong that found that the prevalence of depression caused by COVID-19 was 49.8% [9].

Mental disorders, including depression, anxiety, insomnia, and distress, caused by COVID-19 were assessed in our study by Chinese versions of validated measurement tools [24,25,26,27]: PHQ-9, GAD-7, and ISI-7. In our study, the Cronbach’s alpha coefficient of our questionnaire is 0.97. The Cronbach’s alpha coefficients of the Chinese versions of PHQ-9, GAD-7, ISI-7 and IES-7 were 0.920, 0.945, 0.879 and 0.909, respectively, proving these scales have good reliabilities and validities in the application of evaluating mental disorders among Chinese worker with income losses. By reviewing the literature, we found that these Chinese scales are widely used in the study of psychological problems. Especially recently, these four scales have been used to study COVID-19. For example, researchers used them to assess the magnitude of mental health outcomes among healthcare workers treating patients exposed to COVID-19 in China [10], PHQ-9 and GAD-7 were used to evaluate depression and anxiety in Hong Kong during the COVID-19 pandemic [9], and GAD-7 was used to assess the prevalence of mental health problems and examine their association with social media exposure [8].

In this study, besides age, sex and other demographic characteristics, participants from Hubei province and outside Hubei province were taken as the research objects for comparison of regional differences. The proportions of respondents from Hubei province and places outside Hubei province were 44.2% and 55.8%, respectively. The proportions of light, middle, and heavy losses of income (>0 to 25%, 25–50%, and >50% less income than pre-epidemic levels, respectively) caused by COVID-19 were 33.9%, 17.6%, and 48.5%, respectively. Most of these participants were aged from 26 to 40 years, lived in urban areas, and had a college degree or above. We found that workers with heavy income losses caused by COVID-19 reported more symptoms of depression, anxiety, and insomnia. Compared with participants outside Hubei province, those in Hubei province reported higher scores on all four scales. The prevalence rates of our participants who had severe mental symptoms of depression, anxiety, insomnia, and distress were 19.1%, 21.9%, 7.8%, and 25.9%, respectively. Our findings further indicated that pregnant women scored higher than non-pregnant women on PHQ-9 and GAD-7 measuring symptoms of depression and anxiety. These findings are consistent with the previous studies’ findings that exposure to a public health emergency can cause mental health problems.

This study has several limitations. First, it was limited in scope. Almost half of the participants (44.2%) were from Hubei province, limiting the generalization of our findings to less affected regions. This survey was mainly conducted online, so some respondent bias, such as few elder citizens’ participation, may have affected the results. Second, the survey was conducted over two weeks and lacked longitudinal follow-up. It was hard to determine whether the mental health symptoms of workers with income losses could become more severe, so the long-term psychological implications of this population are worth further investigation. Last, although the response rate of this study was 70.1%, response bias may still exist if the non-respondents were either too stressed to respond or not at all stressed and therefore not interested in this survey.

## 5. Conclusions

In conclusion, our findings showed that relatively high prevalence rates of symptoms of depression, anxiety, insomnia, and distress were caused by COVID-19. The prevalence of mental health problems among workers caused by COVID-19 in China is high, especially those working in Hubei province with heavy income losses. In addition, pregnant women with income losses were associated with a greater risk of feeling depressed and anxiety than other women, and may need psychological support or interventions. These results further indicate that the long-term psychological implications of this population are worth further investigation.

## Figures and Tables

**Table 1:** Demographic and occupational characteristics of participants.

Demographics	Total (%)	Income Losses Caused by COVID-19		
		Light	Middle	Heavy
	398 (100%)	135 (33.9%)	70 (17.6%)	193 (48.5%)
Age (years)				
18–25	78 (19.6%)	31 (7.8%)	10 (2.5%)	37 (9.3%)
26–30	127 (31.9%)	40 (10.1%)	14 (3.5%)	73 (18.3%)
31–40	145 (36.4%)	44 (11.1%)	38 (9.5%)	63 (15.8%)
>40	48 (12.1%)	20 (5.0%)	8 (2.0%)	20 (5.0%)
Sex				
Male	201 (50.5%)	67 (16.8%)	39 (9.8%)	95 (23.9%)
Female	197 (49.5%)	68 (17.1%)	31 (7.8%)	98 (24.6%)
Education				
<Undergraduate	110 (27.6%)	22 (5.5%)	17 (4.3%)	71 (17.8%)
College	180 (45.2%)	66 (16.6%)	42 (10.6%)	72 (18.1%)
>Master	108 (27.1%)	47 (11.8%)	11 (2.8%)	50 (12.6%)
Marital Status				
Married	197 (49.5%)	72 (18.1%)	32 (8.0%)	93 (23.4%)
Not married	201 (50.5%)	63 (15.8%)	38 (9.5%)	100 (25.1%)
Working Location				
Hubei Province	176 (44.2%)	43 (10.8%)	30 (7.5%)	103 (25.9%)
Others	222 (55.8%)	92 (23.1%)	40 (10.1%)	90 (22.6%)
Residence				
Urban	298 (74.9%)	116 (29.1%)	51 (12.8%)	131 (32.9%)
Rural	100 (25.1%)	19 (4.8%)	19 (4.8%)	62 (15.6%)

**Table 2:** Prevalence of depression and associated factors.

Variable	Prevalence	aOR (95% CI)	*p*-Value	
			Category	Overall
PHQ-9, depression symptoms				
Overall	181/398 (45.5%)			
Age (years)				
18–25	32/78 (41.0%)	0.187 (0.072, 0.489)	0.001	<0.001
26–30	45/127 (35.4%)	0.228 (0.097, 0.535)	0.001	
31–40	69/145 (47.5%)	0.597 (0.275, 1.294)	0.191	
>40	35/48 (72.9%)	1 (Reference)	NA	
Sex				
Male	93/201 (46.3%)	1.013 (0.641, 1.605)	0.956	0.956
Female	88/197 (44.7%)	1 (Reference)	NA	
Education				
<Undergraduate	67/110 (60.9%)	1.640 (0.846, 3.178)	0.143	0.336
College	76/180 (42.2%)	1.292 (0.741, 2.252)	0.366	
>Master	38/108 (35.2%)	1 (Reference)	NA	
Marital Status				
Married	113/197 (57.4%)	3.348 (1.896, 5.911)	<0.001	<0.001
Not married	68/201 (33.8%)	1 (Reference)	NA	
Working Location				
Hubei Province	108/176 (61.4%)	2.647 (1.662, 4.217)	<0.001	<0.001
Others	73/222 (32.9%)	1 (Reference)	NA	
Residence				
Urban	112/298 (37.6%)	0.391 (0.226, 0.675)	0.001	0.001
Rural	69/100 (69.0%)	1 (Reference)	NA	
Income losses caused by COVID-19				
Light	32/135 (23.7%)	0.215 (0.124, 0.371)	<0.001	<0.001
Middle	30/70 (49.2%)	0.429 (0.231, 0.797)	0.007	
Heavy	119/193 (61.7%)	1 (Reference)	NA	

Table 2 lists the detailed results of PHQ-9 from multivariable logistic regression analysis; the results for the other scales are presented in Supplementary Materials (Tables S1–S3). Abbreviations: NA = Not Available; aOR: adjusted odds ratio; CI: confidence interval. PHQ-9: the Patient Health Questionnaire-9.

**Table 3:** Prevalence rates of mental symptoms and associated factors in female cohort.

Variable	Prevalence	aOR (95% CI)	*p*-Value	
			Category	Overall
PHQ-9, depression symptoms				
Pregnancy				
Pregnant	29/45 (64.4%)	2.956 (1.208, 7.229)	0.018	0.018
Not pregnant	64/152 (42.1%)	1 (Reference)	NA	
GAD-7, anxiety symptoms				
Pregnancy				
Pregnant	37/45 (82.8%)	3.146 (1.217, 6.133)	0.018	0.018
Not pregnant	75/152 (49.3%)	1 (Reference)	NA	
ISI, insomnia symptoms				
Pregnancy				
Pregnant	24/45 (53.3%)	1.578 (0.654, 3.804)	0.601	0.601
Not pregnant	45/152 (29.6%)	1 (Reference)	NA	
IES-7, distress symptoms				
Pregnancy				
Pregnant	38/45 (84.4%)	1.605 (0.595, 4.328)	0.350	0.350
Not pregnant	102/152 (67.1%)	1 (Reference)	NA	

Abbreviations: NA = Not Available; aOR: adjusted odds ratio; CI: confidence interval. PHQ-9: the Patient Health Questionnaire-9; GAD-7: the Generalized Anxiety Disorder-7; ISI-7: the Insomnia Severity Index-7; IES-7: the revised 7-item Impact of Event Scale.

**Table 4:** Prevalence of severe depression and associated factors.

Variable	No. of Severe Cases/ No. of Total Cases (%)	aOR (95% CI)	*p*-Value	
			Category	Overall
PHQ-9, depression symptoms				
Overall	76/398 (19.1%)			
Age (years)				
18–25	15/78 (19.2%)	0.404 (0.139, 1.175)	0.096	0.042
26–30	16/127 (12.6%)	0.243 (0.091, 0.645)	0.005	
31–40	32/145 (22.1%)	0.450 (0.189, 1.070)	0.071	
>40	13/48 (27.1%)	1 (Reference)	NA	
Sex				
Male	37/201 (18.4%)	0.864 (0.500, 1.495)	0.601	0.601
Female	39/197 (19.8%)	1 (Reference)	NA	
Education				
<Undergraduate	38/110 (34.5%)	1.369 (0.646, 2.899)	0.413	0.060
College	20/180 (11.1%)	0.590 (0.285, 1.219)	0.154	
>Master	18/108 (16.7%)	1 (Reference)	NA	
Marital Status				
Married	38/197 (19.3%)	1.539 (0.826, 2.868)	0.173	0.173
Not married	38/201 (18.9%)	1 (Reference)	NA	
Working Location				
Hubei Province	42/176 (23.9%)	1.085 (0.619, 1.903)	0.776	0.776
Others	34/222 (15.3%)	1 (Reference)	NA	
Residence				
Urban	40/298 (13.4%)	0.337 (0.185, 0.615)	<0.001	<0.001
Rural	36/100 (36.0%)	1 (Reference)	NA	
Income losses caused by COVID-19				
Light	12/135 (8.9%)	0.246 (0.121, 0.502)	<0.001	<0.001
Middle	3/70 (4.3%)	0.086 (0.025, 0.291)	<0.001	
Heavy	61/193 (31.6%)	1 (Reference)	NA	

Table 4 lists the detailed results of PHQ-9 from multivariable logistic regression analysis; the results for the other scales are presented in Supplementary Materials (Tables S4–S6). Abbreviations: NA = Not Available; aOR: adjusted odds ratio; CI: confidence interval. PHQ-9: the Patient Health Questionnaire-9.

## Supplementary Materials

The following are available online at https://www.mdpi.com/1660-4601/17/15/5627/s1, Table S1: Prevalence of anxiety and associated factors, Table S2: Prevalence of insomnia and associated factors, Table S3: Prevalence of distress and associated factors, Table S4: Prevalence of severe anxiety and associated factors, Table S5: Prevalence of severe insomnia and associated factors, Table S6: Prevalence of severe distress and associated factors.

## Appendix A

The questionnaire consisted of 37 questions to record demographic indicators and symptoms of depression, anxiety, insomnia, and distress of all participants.

1. Demographic data

The following demographic data were included in this study: sex (male or female), age (18–25, 26–30, 31–40, or >40 years categories), educational level (<undergraduate, college, masters, or higher), marital status (recoded into married or other including unmarried, widowed, and divorced), working location (Hubei province or outside Hubei province), loss of income caused by COVID-19 (light, middle, and heavy, being >0 to 25%, 25–50%, and >50% less income than the pre-epidemic level, respectively), and place of residence (urban or rural).

2. Mental Health Scales

The English versions of the PHQ-9, GAD-7, ISI-7, and IES–R-7 scales were used in this study to measure the degree of symptoms of depression, anxiety, insomnia, and distress of our participants.

**Table A1:** Patient Health Questionnaire-9 (PHQ-9).

Depression Problem	Not at All	Somewhat	More than Half the Days	Nearly Every Day
Little interest or pleasure in doing things?	0	1	2	3
Feeling down, depressed, or hopeless?	0	1	2	3
Trouble falling or staying asleep, or sleeping too much?	0	1	2	3
Feeling tired or having little energy?	0	1	2	3
Poor appetite or overeating?	0	1	2	3
Feeling bad about yourself or that you are a failure or have let yourself or your family down?	0	1	2	3
Trouble concentrating on things, such as reading the newspaper or watching television?	0	1	2	3
Moving or speaking so slowly that other people could have noticed? Or so fidgety or restless that you have been moving a lot more than usual?	0	1	2	3
Thoughts that you would be better off dead, or thoughts of hurting yourself in some way?	0	1	2	3
Total				

**Table A2:** General Anxiety Disorder-7 (GAD-7).

Anxiety Problem	Not at All	Several	More than Half the Days	Nearly Every Day
Feeling nervous, anxious, or on edge	0	1	2	3
Not being able to stop or control worrying	0	1	2	3
Worrying too much about different things	0	1	2	3
Trouble relaxing	0	1	2	3
Being so restless that it’s hard to sit still	0	1	2	3
Becoming easily annoyed or irritable	0	1	2	3
Feeling afraid as if something awful might happen	0	1	2	3
Total				

**Table A3:** Insomnia Severity Index-7 (ISI-7).

Insomnia Problem	None	Mild	Moderate	Severe	Very Severe
Difficulty falling asleep	0	1	2	3	4
Difficulty staying asleep	0	1	2	3	4
Problems waking up too early	0	1	2	3	4
How satisfied/dissatisfied are you with your current sleep pattern?	0	1	2	3	4
How noticeable to others do you think your sleep problem is in terms of impairing the quality of your life?	0	1	2	3	4
How worried/ distressed are you about your current sleep problem?	0	1	2	3	4
To what extent do you consider your sleep problem to interfere with your daily functioning currently?	0	1	2	3	4
Total					

**Table A4:** Revised Event Impact Scale (IES-7).

Distress Problem	None	Mild	Moderate	Severe	Very Severe
It’s upsetting to think about COVID-19	0	1	2	3	4
COVID-19 has affected my quality of life	0	1	2	3	4
COVID-19 has affected my learning/work/income/employment	0	1	2	3	4
COVID-19 has affected family/couple relations	0	1	2	3	4
I dream about COVID-19	0	1	2	3	4
I try not to mention anything about COVID-19	0	1	2	3	4
I am still worried about COVID-19	0	1	2	3	4
Total					
