# Supplementary material for: Factors Associated with Mental Health Results among Workers with Income Losses Exposed to COVID-19 in China
Source: Int J Environ Res Public Health. 2020 Aug 4;17(15):5627. doi: 10.3390/ijerph17155627 (PMC7432610; doi:10.3390/ijerph17155627)
Supplement: Supplementary file 1 [file ijerph-17-05627-s001.pdf]

Table S1. Prevalence of anxiety and associated factors.

Variable	Prevalence	aOR (95% CI)	*p*-Value	
			Category	Overall
GAD-7, anxiety symptoms				
Overall	197/398 (49.5%)			
Age (years)				
18–25	33/78 (42.3%)	0.357 (0.144, 0.888)	0.027	<0.001
26–30	40/127 (31.5%)	0.284 (0.126, 0.639)	0.002	
31–40	99/145 (68.3%)	1.429 (0.687, 2.975)	0.340	
>40	25/48 (52.1%)	1 (Reference)	NA	
Sex				
Male	97/201 (48.3%)	0.891 (0.576, 1.380)	0.606	0.606
Female	100/197 (50.8%)	1 (Reference)	NA	
Education				
<Undergraduate	68/110 (61.8%)	1.128 (0.473, 1.662)	0.708	0.111
College	74/180 (41.1%)	0.576 (0.279, 0.992)	0.006	
>Master	55/108 (50.9%)	1 (Reference)	NA	
Marital Status				
Married	104/197 (52.8%)	1.520 (0.902, 2.559)	0.116	0.116
Not married	93/201 (46.2%)	1 (Reference)	NA	
Working Location				
Hubei Province	121/176 (68.8%)	3.562 (2.285, 5.552)	<0.001	<0.001
Others	76/222 (34.2%)	1 (Reference)	NA	
Residence				
Urban	130/298 (43.6%)	0.468 (0.276, 0.796)	0.005	0.005
Rural	67/100 (67.0%)	1 (Reference)	NA	
Income losses caused by COVID-19				
Light	56/135 (41.5%)	0.605 (0.365, 1.001)	<0.001	0.001
Middle	24/70 (34.3%)	0.316 (0.167, 0.595)	0.008	
Heavy	117/193 (60.6%)	1 (Reference)	NA	

Table S2. Prevalence of insomnia and associated factors.

Variable	Prevalence	aOR (95% CI)	*p*-value	
			Category	Overall
ISI, insomnia symptoms				
Overall	123/398 (30.9%)			
Age (years)				
18–25	16/78 (20.5%)	0.385 (0.141, 1.053)	0.063	0.001
26–30	22/127 (17.3%)	0.328 (0.135, 0.797)	0.014	
31–40	69/145 (47.6%)	1.210 (0.565, 2.592)	0.624	
>40	16/48 (33.3%)	1 (Reference)	NA	
Sex				
Male	56/201 (27.9%)	0.685 (0.421, 1.115)	0.128	0.128
Female	67/197 (34.0%)	1 (Reference)	NA	
Education				
<Undergraduate	57/110 (51.8%)	1.046 (0.536, 2.043)	0.894	0.703
College	34/180 (18.9%)	0.779 (0.504, 1.103)	0.802	
>Master	32/108 (29.6%)	1 (Reference)	NA	
Marital Status				
Married	51/197 (25.9%)	0.984 (0.564, 1.716)	0.955	0.955
Not married	72/201 (35.8%)	1 (Reference)	NA	
Working Location				
Hubei Province	79/176 (44.9%)	2.347 (1.441, 3.823)	0.001	0.001
Others	44/222 (19.8%)	1 (Reference)	NA	

Residence				
Urban	64/298 (21.5%)	0.210(0.122, 0.363)	<0.001	<0.001
Rural	59/100 (59.0%)	1 (Reference)	NA	
Income losses caused by COVID-19				
Light	28/135 (20.7%)	0.497 (0.279, 0.883)	0.017	0.001
Middle	14/70 (20.0%)	0.314 (0.153, 0.644)	0.001	
Heavy	81/193 (42.0%)	1 (Reference)	NA	

Table S3. Prevalence of distress and associated factors.

Variable	Prevalence	aOR (95% CI)	*p*-value	
			Category	Overall
IES-7, distress symptoms				
Overall	271/398 (30.9%)			
Age (years)				
18–25	46/78 (59.0%)	0.300 (0.114, 0.789)	0.015	<0.001
26–30	66/127 (52.0%)	0.262 (0.111, 0.615)	0.002	
31–40	121/145 (83.4%)	1.039 (0.442, 2.440)	0.930	
>40	38/48 (79.2%)	1 (Reference)	NA	
Sex				
Male	135/201 (67.2%)	0.949 (0.605, 1.487)	0.818	0.818
Female	136/197 (69.0%)	1 (Reference)	NA	
Education				
<Undergraduate	76/110 (69.1%)	0.824 (0.438, 1.551)	0.548	0.191
College	129/180 (71.7%)	1.394 (0.819, 2.372)	0.221	
>Master	66/108 (61.1%)	1 (Reference)	NA	
Marital Status				
Married	136/197 (69.0%)	1.093 (0.638, 1.874)	0.745	0.745
Not married	135/201 (67.2%)	1 (Reference)	NA	
Working Location				
Hubei Province	143/176 (81.3%)	2.950 (1.815, 4.794)	<0.001	<0.001
Others	128/222 (57.7%)	1 (Reference)	NA	
Residence				
Urban	192/298 (64.4%)	0.540(0.302, 0.965)	0.033	0.033
Rural	79/100 (79.0%)	1 (Reference)	NA	
Income losses caused by COVID-19				
Light	90/135 (66.7%)	1.176 (0.701, 1.974)	0.538	0.809
Middle	48/70 (68.6%)	1.009 (0.535, 1.903)	0.979	
Heavy	133/193 (68.9%)	1 (Reference)	NA	

Table S4. Prevalence of severe anxiety and associated factors.

Variable	No. of Severe Cases/ No. of Total Cases (%)	OR [a] (95% CI [b])	*p*-Value	
			Category	Overall
GAD-7, anxiety symptoms				
Overall	87/398 (21.9%)			
Age (years)				
18–25	9/78 (11.5%)	0.200 (0.065, 0.617)	0.005	0.001
26–30	15/127 (11.8%)	0.216 (0.083, 0.563)	0.002	
31–40	43/145 (29.6%)	0.715 (0.322, 1.588)	0.410	
>40	20/48 (41.7%)	1 (Reference)	NA	
Sex				
Male	36/201 (17.9%)	0.593 (0.346, 1.019)	0.057	0.057
Female	51/197 (25.9%)	1 (Reference)	NA	
Education				
<Undergraduate	42/110 (38.2%)	0.970 (0.458, 2.052)	0.936	0.018

College	24/180 (13.3%)	0.429 (0.212, 0.871)	0.019	
>Master	21/108 (19.4%)	1 (Reference)	NA	
Marital Status				
Married	44/197 (22.3%)	1.119 (0.610, 2.053)	0.716	0.716
Not married	43/201 (21.4%)	1 (Reference)	NA	
Working Location				
Hubei Province	60/176 (34.1%)	2.570(1.478,4.466)	0.001	0.001
Others	27/222 (12.2%)	1 (Reference)	NA	
Residence				
Urban	47/298 (15.8%)	0.385 (0.213, 0.696)	0.002	0.002
Rural	40/100 (40.0%)	1 (Reference)	NA	
Income losses caused by COVID-19				
Light	15/135 (11.1%)	0.332 (0.168, 0.658)	0.002	<0.001
Middle	9/70 (12.9%)	0.278 (0.123, 0.629)	0.001	
Heavy	63/193 (32.6%)	1 (Reference)	NA	

Table S5. Prevalence of severe insomnia and associated factors.

Variable	No. of Severe Cases/<br>No. of Total Cases (%)	aOR [a] (95% CI [b])	*p*-Value	
			Category	Overall
ISI, insomnia symptoms				
Overall	31/398 (7.8%)			
Age (years)				
18–25	4/78 (5.1%)	0.613 (0.125, 3.005)	0.546	0.423
26–30	4/127 (3.1%)	0.356 (0.080, 1.576)	0.173	
31–40	18/145 (12.4%)	0.902 (0.289, 2.810)	0.858	
>40	5/48 (10.4%)	1 (Reference)	NA	
Sex				
Male	15/201 (7.5%)	0.937 (0.429, 2.045)	0.870	0.870
Female	16/197 (8.1%)	1 (Reference)	NA	
Education				
<Undergraduate	18/110 (16.4%)	2.304 (0.653, 8.130)	0.194	0.250
College	9/180 (5.0%)	1.100 (0.321, 3.772)	0.879	
>Master	4/108 (3.7%)	1 (Reference)	NA	
Marital Status				
Married	11/197 (5.6%)	0.784 (0.326, 1.886)	0.585	0.585
Not married	20/201 (10.0%)	1 (Reference)	NA	
Working Location				
Hubei Province	21/176 (11.9%)	1.791(0.763,4.202)	0.180	0.180
Others	10/222 (4.5%)	1 (Reference)	NA	
Residence				
Urban	14/298 (4.7%)	0.385 (0.163, 0.906)	0.030	0.030
Rural	17/100 (17.0%)	1 (Reference)	NA	
Income losses caused by COVID-19				
Light	6/135 (4.4%)	0.589 (0.209, 1.658)	0.589	0.107
Middle	2/70 (2.9%)	0.251 (0.055, 1.142)	0.251	
Heavy	23/193 (11.9%)	1 (Reference)	NA	

Table S6. Prevalence of severe distress and associated factors.

Variable	No. of Severe Cases/<br>No. of Total Cases (%)	OR [a] (95% CI [b])	*p*-Value	
			Category	Overall
IES-7, distress symptoms				
Overall	103/398 (25.9%)			
Age (years)				
18–25	11/78 (14.1%)	0.195 (0.063, 0.597)	0.004	<0.001
26–30	13/127 (10.2%)	0.159 (0.059, 0.433)	<0.001	

31–40	63/145 (43.4%)	0.921 (0.409, 2.073)	0.842	
>40	16/48 (33.3%)	1 (Reference)	NA	
Sex				
Male	50/201 (24.9%)	0.911 (0.531, 1.561)	0.734	0.734
Female	53/197 (26.9%)	1 (Reference)	NA	
Education				
<Undergraduate	53/110 (48.2%)	1.595 (0.751, 3.386)	0.224	0.017
College	31/180 (17.2%)	0.610 (0.302, 1.234)	0.169	
>Master	19/108 (17.6%)	1 (Reference)	NA	
Marital Status				
Married	53/197 (26.9%)	1.180 (0.642, 2.170)	0.593	0.593
Not married	50/201 (24.9%)	1 (Reference)	NA	
Working Location				
Hubei Province	76/176 (43.2%)	4.066(2.328,7.102)	<0.001	<0.001
Others	27/222 (12.2%)	1 (Reference)	NA	
Residence				
Urban	50/298 (16.8%)	0.227 (0.126, 0.410)	<0.001	<0.001
Rural	53/100 (53.0%)	1 (Reference)	NA	
Income losses caused by COVID-19				
Light	17/135 (12.6%)	0.328 (0.166, 0.645)	0.001	<0.001
Middle	13/70 (18.6%)	0.325 (0.152, 0.694)	0.004	
Heavy	73/193 (37.8%)	1 (Reference)	NA	
